# Host plants and obligate endosymbionts are not the sources for biosynthesis of the aphid alarm pheromone

**DOI:** 10.1038/s41598-017-06465-9

**Published:** 2017-07-20

**Authors:** Zhi-Juan Sun, Zheng-Xi Li

**Affiliations:** Department of Entomology, China Agricultural University, 2 Yuanmingyuan West Road, Beijing, 100193 China

## Abstract

(*E*)-β-farnesene (EβF) is the major component of the alarm pheromone of many aphid species, but where EβF is synthesized in aphids is only partly understood. There are at least three most possible sources for the alarm pheromone: host plants, aphid obligate endosymbiont and aphids themselves. Here we eliminated the possibility of host plants and the obligate endosymbiont *Buchnera aphidicola* as the sources for EβF released by aphids. We excluded the possible effects of host plants on EβF biosynthesis by rearing aphids on non-plant diets. Both the diet-reared aphids, including the cotton aphid *Aphis gossypii* and the green peach aphid *Myzus persicae*, could still release EβF based on solid-phase micro-extraction combined with gas chromatography-mass spectrometer analysis. Meanwhile, we treated host aphids with antibiotics to fully eliminate *Buchnera* bacteria. Though the treatment seriously affected the development and fecundity of host aphids, the treated aphids could still release EβF, and there was no significant difference in the EβF concentration as per the aphid weight under different rearing conditions. Taken together, our experimental results suggest that host plants and obligate endosymbionts are not the sources for EβF released by aphids, indicating that it is most probably the aphid itself synthesizes the alarm pheromone.

## Introduction

Aphids are among the most abundant and destructive insect pests, which damage plants not only by sucking phloem sap, secreting honeydew to develop plant sooty moulds, but by transmitting plant viruses^1^. The annual worldwide crop losses due to aphids are estimated at hundreds of millions of dollars^2^. Most aphids will release alarm pheromone to warn their conspecifics of potential threats when attacked by natural enemies, and the main component of the alarm pheromone of many aphid species is the sesquiterpene, (*E*)-β-farnesene (EβF)^3–5^, which is secreted by cornicles and has been used for control of aphids^3, 6, 7^. Unfortunately, up to now, where EβF is synthesized in aphids is only partly understood.

Almost all terpenes are derived from the precursors synthesized by successive condensation of dimethylallyl diphosphate (DMAPP, C_5_) with different units of isopentenyl diphosphate (IPP, C_5_)^8, 9^. The short-chain prenyl diphosphate products include geranyl diphosphate (GPP, C_10_), farnesyl diphosphate (FPP, C_15_), and geranylgeranyl diphosphate (GGPP, C_20_)^10, 11^, which are the common precursors of monoterpenes, sesquiterpenes, and diterpenes, respectively^12^. The condensation reactions are catalyzed by geranyl diphosphate synthase (GPPS), farnesyl diphosphate synthase (FPPS), and geranylgeranyl diphosphate synthase (GGPPS) ^13^, respectively. In insects, FPPSs have been intensively studied^14–17^ because of their involvement in the biosynthetic pathway of the sesquiterpenoid juvenile hormone (JH)^16, 18^. EβF and JH share the C_15_ precursor, FPP. In recent years, FPPSs have been characterized *in vitro* in different aphid species^19–23^, showing that this enzyme can utilize IPP and DMAPP to produce FPP with GPP as a transient product. In our laboratory, we cloned and characterized five full-length cDNA sequences of FPPS from different aphids, and found that the cotton aphid *Aphis gossypii* contains only one FPPS gene^20^, while both the green peach aphid *Myzus persicae* and the bird cherry-oat aphid *Rhopalosiphum padi* have two FPPS genes ^23, 24^.

The whole genome sequence of the pea aphid *Acyrthosiphon pisum* has become available^25^, but no sequences displaying characteristic motifs of the terpene-synthase family have been identified^26^. Interestingly, in plants, cDNAs encoding the EβF synthase have been isolated from *Mentha piperita* ^27^, *Zea mays* ^28^, *Citrus junos* ^29^ and *Artemisia annua* ^30^. Some of these EβF synthase genes have been engineered into target plants, and the latter could release EβF with an implication in aphid control^31–33^. The plant-originated EβF synthase was successfully used in combination with aphid FPPS to produce EβF *in vitro* ^19^, indicating that the aphid FPPS might be involved in the biosynthesis of aphid alarm pheromone. Unfortunately, up to date, no genes responsible for biosynthesis of EβF in aphids have been identified, nor EβF has been synthesized exclusively based on aphid genes *in vitro*.

The isoprenoid pathway has also been studied in microorganisms. Fujisaki *et al*.^34^ found that the geranyl transferase *ispA* gene was responsible for the biosynthesis of FPP in *Escherichia coli*. The whole genome sequence of the obligate intracellular endosymbiont of aphid, *Buchnera aphidicola*, which provides essential amino acids and other nutrients for its hosts^35^ also contains *ispA* gene^36–38^, though its function has not been characterized. The functional plasticity of terpenoid synthases was reported in plants, insects and microbes. For instances, Green *et al*.^39^ found apple α-farnesene synthase possessing prenyltransferase activity; the isoprenyl diphosphate synthase in the bark beetle *Ips pini* had an amateur function of terpene synthase^40, 41^, while the monooxygenase albaflavenone synthase in the soil bacterium, *Streptomyces coelicolor*, was shown to have a moonlighting terpene synthase activity^42^.

Since the genes encoding the enzymes responsible for the biosynthesis of EβF have not been identified in the aphid genome, we thus speculate that there are at least three possible sources for EβF biosynthesis: host plants, obligate endosymbionts, and aphids themselves. A previous work demonstrated that the aphids (*M. persicae*) reared on standard artificial diet could release EβF though the sizes of these diet-reared aphids became smaller, but an acetate-supplemented diet could rescue this defect^43^. In the present study, we used a modified diet formula and two aphid species, *A. gossypii* and *M. persicae*, to confirm that both an artificial diet and the obligate endosymbionts could not prevent EβF production in aphids, based on our solid-phase micro-extraction (SPME) combined with gas chromatography-mass spectrometry (GC-MS) results.

## Results

### Effects of artificial diets on the development and fecundity of aphids

The aphids *M. persicae* and *A. gossypii* were reared on improved diets modified from the published prescription^44^. The results for *M. persicae* and *A. gossypii* were totally different: *M. persicae* could not go down beyond the third generation (F_3_) (only a few sterile offspring was seen), while *A. gossypii* went down to F_23_ at this point. Our experiments showed that the developmental duration of the first-instar *M. persicae* nymphs of the first generation (F_1_) reared on artificial diet was significantly longer than that of *M. persicae* reared on pepper plants (*P* < 0.01), while the developmental durations of the diet-reared second-, third- and fourth-instar nymphs were not significantly different from those of pepper-reared ones (*P* = 0.198, 0.581 and 0.771, respectively) (Table 1). The development of diet-reared *M. persicae* adults was obviously delayed compared with that of pepper-reared counterparts (*P* < 0.01). As a whole, the diet-reared *M. persicae* aphids lived highly significantly longer than pepper-reared ones (*P* < 0.01), and their fecundity was also extremely significantly decreased compared to that of pepper-reared *M. persicae* aphids (*P* < 0.01).

**Table 1:** Effects of artificial diets and antibiotic treatment on the development and fecundity of *M. persicae* (F_1_).

	N_1_/d	N_2_/d	N_3_/d	N_4_/d	Adult/d	Longevity/d	Fecundity
Diet	3.67 ± 0.97Aa	2.45 ± 0.47Bb	2.36 ± 0.48Bb	2.61 ± 0.70Bb	24.52 ± 5.16A	35.60 ± 5.08Aa	5.08 ± 2.01B
Pepper	2.73 ± 0.58B	2.10 ± 0.56Bb	2.08 ± 0.64Bb	2.76 ± 0.90Bb	15.04 ± 1.91Bb	24.71 ± 2.16B	35.7 ± 6.81A
Anti-diet	3.86 ± 1.20Aa	4.37 ± 1.25A	6.64 ± 2.66A	7.76 ± 2.68A	12.25 ± 5.61Bb	34.88 ± 5.67Aa	0

Data are mean ± SE. Different lowercase and uppercase letters within the same column indicate significant difference at 5% and 1% levels by using LSD multiple comparison method. Diet: Artificial diet; Anti-diet: Diet containing antibiotics.

Unlike *M. persicae*, *A. gossypii* reared on artificial diets exhibited no significant difference from cotton-reared aphids in the developmental duration over different developmental stages from the fifth generation on (N_1_, *P* = 0.453; N_2_, *P* = 0.285; N_3_, *P* = 0.127; N_4_, *P* = 0.139; adult, *P* = 0.761) (Table 2; data for F_17_), although the fecundity of diet-reared *A. gossypii* was significantly decreased compared with cotton-reared aphids (*P* < 0.01). Considering that *A. gossypii* lived much better on the artificial diets than *M. persicae*, *A. gossypii* aphids (F_17_) and their offspring reared on artificial diets, antibiotic-treated diets and plants were sampled for volatile analysis to examine the effects of host plants and endosymbionts on the biosynthesis of EβF in the present study.

**Table 2:** Effects of artificial diets and antibiotic treatment on the development and fecundity of *A. gossypii* (F_17_). Data are mean ± SE.

	N_1_/d	N_2_/d	N_3_/d	N_4_/d	Adult/d	Longevity/d	Fecundity
Diet	2.30 ± 0.71a	2.14 ± 0.46Bb	2.25 ± 0.55Bb	2.81 ± 0.42Bb	33.77 ± 6.73Aa	43.26 ± 6.44a	29.40 ± 4.03B
Cotton	2.17 ± 0.30a	1.90 ± 0.38Bb	1.83 ± 0.35Bb	2.34 ± 0.39Bb	34.46 ± 6.07Aa	42.70 ± 6.25ab	42.35 ± 7.77A
Anti-diet	2.52 ± 0.60a	3.67 ± 1.03A	5.03 ± 1.31A	6.00 ± 1.61A	20.90 ± 8.36B	38.12 ± 8.90b	0

Different lowercase and uppercase letters within the same column indicate significant difference at 5% and 1% levels by using LSD multiple comparison method. Diet: Artificial diet; Anti-diet: Diet containing antibiotics.

### Elimination of *Buchnera* and its effects on aphid development and fecundity

**Figure 1:**
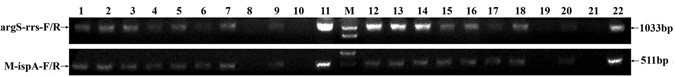
Elimination of *Buchnera* bacteria from aphids. 1–10: Fed on tetracycline-treated diet for 2, 2, 6, 6, 10, 10, 12, 12, 14 and 14 days before fed on non-antibiotic diet for 5, 10, 5, 10, 5, 10, 5, 10, 5 and 10 days, respectively; 12–21: Fed on rifampicin-treated diet for 2, 2, 6, 6, 10, 10, 12, 12, 14 and 14 days before fed on non-antibiotic diet for 5, 10, 5, 10, 5, 10, 5, 10, 5 and 10 days, respectively. PCR products were amplified by using the primers argS-rrs-F/R and M-*ispA*-F/R (cropped from Supplementary Figure S1). Marker: 1000/750 bp (upper); 750/500 bp (down).

The elimination efficiency of antibiotic treatment against *Buchnera* bacteria was not only dependant on the antibiotic treatment time but also the rearing time on normal diets after antibiotic treatment. As shown (Figs 1 and Supplementary Figure S1), *Buchnera* bacteria in *M. persicae* could be effectively eliminated by reared on normal diets for 10 days in addition to treatment with tetracycline or rifampicin for 12–14 days. Rearing aphids on normal diets for 5 days after antibiotic treatment for 12–14 days could not successfully eliminate *Buchnera* from its host. For molecular detection of *Buchnera in A. gossypii*, we also cloned and sequenced its *ispA* gene (GenBank acc.no. KX999117), which was 847 bp in length and encoded a protein of 282 amino acids. Specific primers (C-*ispA*-F/R) designed based on this sequence was used to detect the existence of *Buchnera* in *A. gossypii*, and the results showed that the same antibiotic treatment protocol could also effectively eliminate *Buchnera* bacteria from *A. gossypii*. Meanwhile, the effects of antibiotic treatment on the development and fecundity were also observed, which indicated that antibiotic diets could fully erase the reproductive ability of both *M. persicae* and *A. gossypii*, whereas its effect on the longevity of *M. persicae* was not significant (*P* = 0.621), but significant on that of *A. gossypii* compared with antibiotic-free diet (*P* = 0.03) (Tables 1 and 2).

### Analysis of volatile releases from aphids by SPME and GC-MS

**Figure 2:**
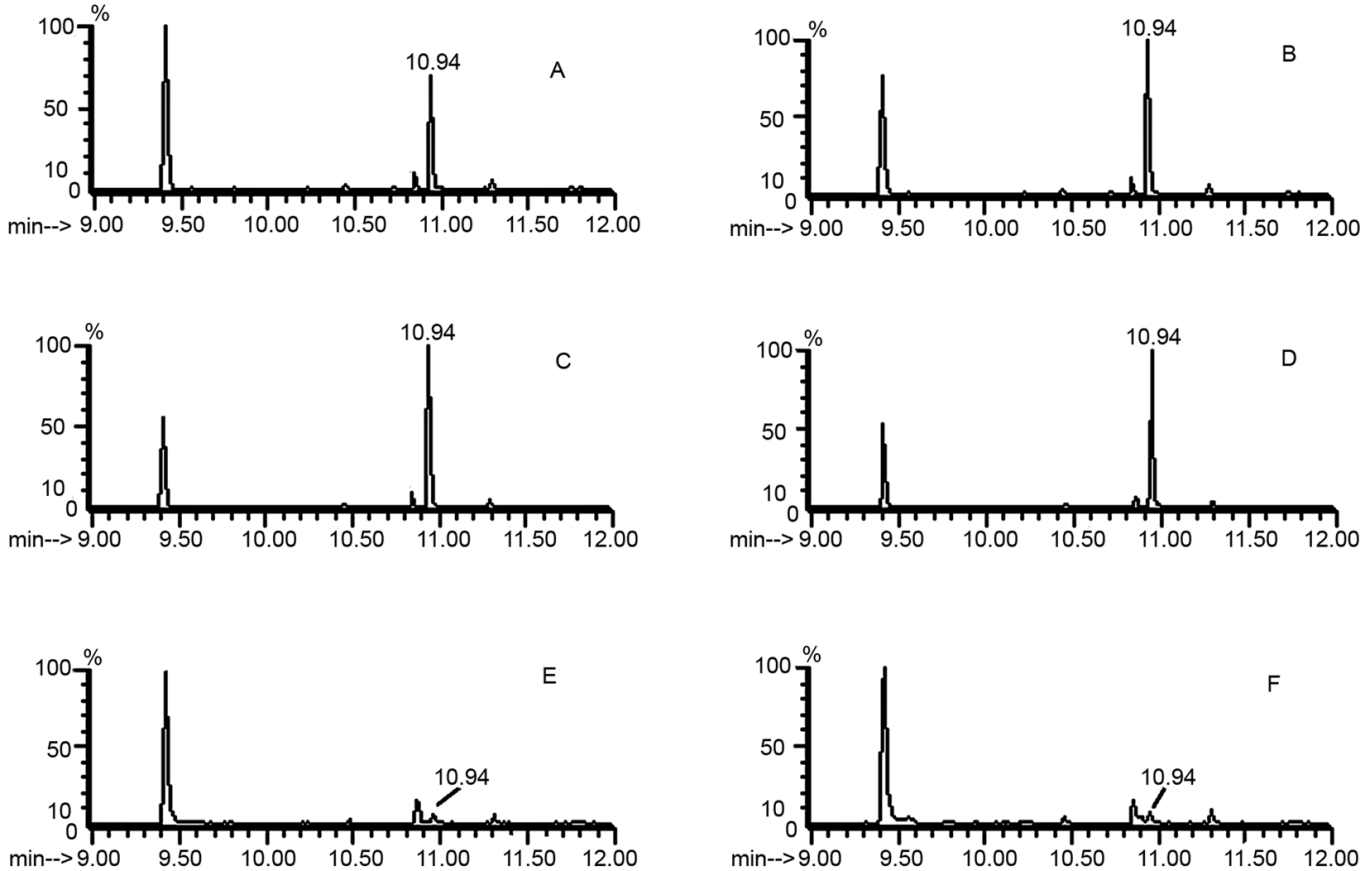
Analysis of the volatiles from aphids under different rearing conditions by GC-MS. (**A**) The volatiles from 10 *A. gossypii* aphids reared on cotton plants. (**B**) The volatiles from 10 *A. gossypii* aphids reared on artificial diets (F_17_). (**C**,**D**) The volatiles from 10 *A. gossypii* aphids treated with antibiotics for 14 days and then reared on antibiotic-free diets for 10 or 22 additional days, respectively. (**E**,**F**) The volatiles from single *A. gossypii* aphid reared on cotton plants and artiicial diets, respectively. The retention time for (*E*)-β- farnesene is 10.94.

The volatiles from diet-reared, antibiotic-treated and cotton-reared *A. gossypii* were analyzed by SPME and GC-MS. The standard solution was a mixture including (*E*)-β-farnesene (EβF) and its isomeride α-farnesene (Supplementary Figure S2A). The retention time for EβF was 10.94, with a molecular weight of 204 (Supplementary Figure S2B), and the retention time for α-farnesene was 10.72. The volatiles from *A.gossypii* reared on cotton plants contained EβF (Fig. 2A), and the aphids reared on artificial diets (F_17_) could also release EβF (Fig. 2B). The aphids treated with antibiotics for 14 days and then reared on antibiotic-free diets for 10 or 22 additional days could still release EβF (Fig. 2C,D). In addition, we could also detect volatile releases from both cotton-reared and diet-reared single aphids of *A. gossypii*, though the amounts of EβF were very small (Fig. 2E,F).

### Quantitative analysis of cornicle secretions

**Figure 3:**
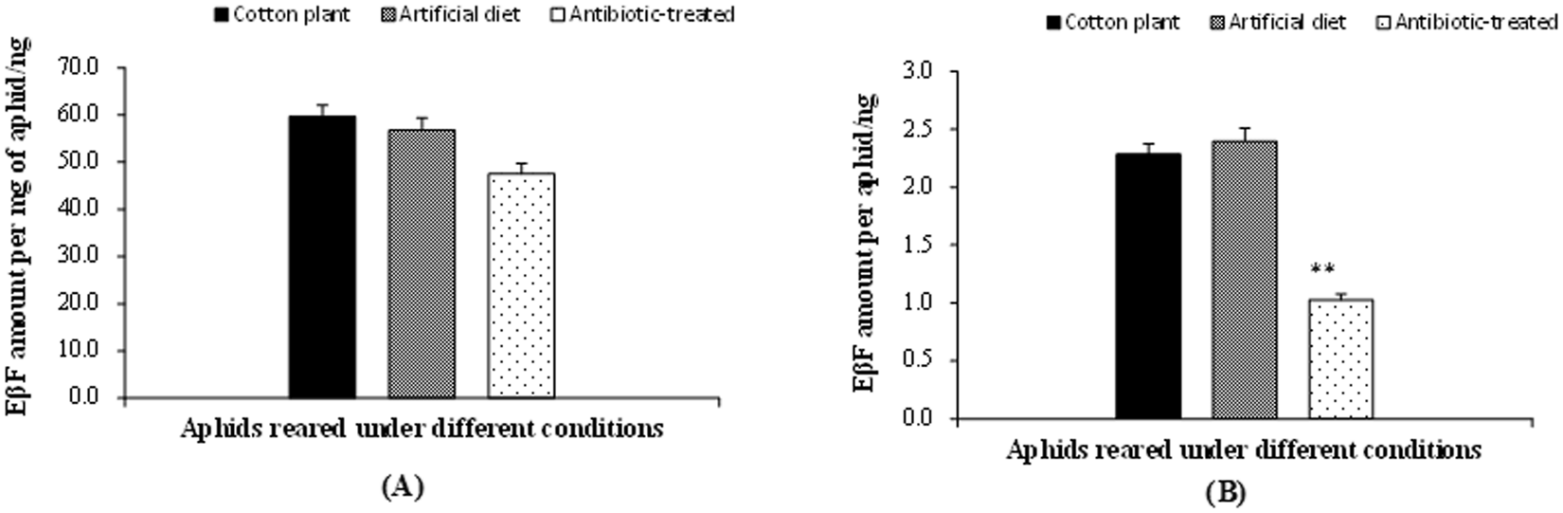
Amount of EβF per mg of aphid (**A**) and per aphid (**B**) under different treatments.

The proportion of aphids emitting cornicle droplets was significantly lower when reared with artificial diet and under antibiotic treatment than that when reared on cotton plants (*P* < 0.001), but the proportion had no significant difference between the aphids treated with and without antibiotics (*P* = 0.123) (Table 3). The mean weight of antibiotic-treated individual aphid was reduced by 50% compared with untreated aphids (*P* < 0.01), but there was no difference between cotton plant-reared and normal diet-reared aphids (*P* = 0.231). The EβF concentration of diet-reared aphid, measured as per milligram of aphid (ng), was not significantly different from those of the plant-reared (*P* = 0.407) and antibiotic-treated aphids (*P* = 0.024), though the mean amount of EβF per mg of antibiotic-treated aphid was reduced by approximately 12 ng compared with that of plant-reared aphids (*P* = 0.006) (Fig. 3A). In addition, the amount of EβF per antibiotic-treated aphid (ng) was significantly less than those of plant-reared (*P* < 0.01) and diet-reared aphid (*P* < 0.01) (Fig. 3B), reduced by approximately 50%, but there was no significant difference between plant-reared and normal diet-reared aphids (*P* = 0.380).

**Table 3:** Proportion of *A. gossypii* emitting cornicle droplets and mean weight of the aphids reared under different treatments.

	Cotton plant	Artificial diet	Antibiotic-treated
Proportion of aphids emitting droplets	0.90 ± 0.01A	0.65 ± 0.02Bb	0.61 ± 0.01Bb
Mean weight (μg)	38.25 ± 1.31Aa	42.25 ± 3.55Aa	21.64 ± 0.46B

Data are mean ± SE. Different lowercase and uppercase letters within the same row indicate significant difference at 5% and 1% levels by using LSD multiple comparison method.

## Discussion

The whole genome sequence of the pea aphid *A. pisum* was published in 2010, but no sequences belonging to the terpene synthase family have been identified. The question is: where and how the aphid alarm pheromone is biologically synthesized? There are at least two other possible sources for biosynthesis of the pheromone: host plants and obligate endosymbionts, since the cDNAs encoding EβF synthase have been sequenced and characterized in plants and microbes^27, 42^. Here, we eliminated the possibility of host plants and the obligate endosymbiont *Buchnera* as the sources for EβF biosynthesis in two aphid species, *A. gossypii* and *M. persicae*, indicating that it is probably the aphids themselves responsible for biosynthesis of their pheromones.

Why is the host plant a possible source for aphid alarm pheromone? It is known that many plant species synthesize and release the sesquiterpene EβF^27–30^. Since no genes responsible for the biosynthesis of EβF have been identified in the aphid even if the whole genome of aphid is available, the possibility for the host plant to be a source of aphid alarm pheromone cannot be excluded. It is not difficult to verify this hypothesis: If the host plant is the source for aphid-released EβF, the aphids reared on non-plant diets would not release EβF. A previous work examining the effect of artificial diet on the production of EβF suggested that the aphids (*M. persicae*) reared on standard artificial diet could release EβF though the sizes of these diet-reared aphids became smaller, which resulted in a decreased total amount of EBF below the alarm response threshold, but an acetate-supplemented diet could rescue this defect^43^. In the present study, our experimental data confirmed that the two aphid species reared on artificial diets could release EβF, which excluded the effects of host plants on the biosynthesis of aphid alarm pheromone, as the ingredients of the diet contained no intermediates or precursors of EβF (Supplementary Table S1). The structural similarity between the major aggregation pheromone components of bark beetles and the monoterpenes found in the host trees led researchers to believe that the insect might use plant-derived precursors to produce their pheromone components^45^, but it is now recognized that most bark beetle aggregation pheromone components are biosynthesized *de novo* ^40, 46^. Nevertheless, the labeling with radiolabeled acetate will provide direct evidence for the biochemical pathway of EBF biosynthesis.

The mutualistic relationship between aphid and its obligate endosymbiont *Buchnera* evolved from about 80–150 million years ago^47, 48^: The aphid hosts *Buchnera*, while *Buchnera* provides its host with essential amino acids and other nutrients^49, 50^. In contrast, aphids are also infected with various facultative endosymbionts which are not essential for aphid development and reproduction. Here we only examined the effect of the obligate endosymbiont *Buchnera* on biosynthesis of EβF in aphids since it is not evolutionarily “safe” for the facultative endosymbionts to be responsible for such an important phenotype as aphid alarm behavior. Considering the functional plasticity of terpenoid synthases, the *ispA* gene encoding FPPS in *Buchnera* may be involved in the production of the sesquiterpene EβF. We thus inactivated *Buchnera* bacteria by antibiotic treatment, and then double checked the bacteria with universal primers and the primers specifically targeting *ispA*. Our results confirmed the validity of our antibiotic treatment procedure, as the subsequent GC-MS analysis verified that the aphids without *Buchnera* bacteria could still release EβF, obviating the possibility of *Buchnera* as a source for biosynthesis of EβF in aphids. The symbiotic bacteria in the New Zealand grass grub beetle were found to be related with the production of the beetle’s sex pheromone^51^, but no direct evidence has been presented. Similarly, the bacterium *Bacillus cereus* isolated from *Ips* bark beetles was shown to be capable of converting α-pinene to verbenol (component of beetle aggregation pheromone) *in vitro* ^52^, but no *in vivo* evidence showing the actual synthesis of verbenol from α-pinene in the beetle gut has been demonstrated.

The diet for rearing *M. persicae* was modified from the published prescription^44^, but *M. persicae* could not live well on it, whereas *A. gossypii* became rapidly adapted to it (we got F_23_ offspring of diet-reared *A. gossypii* at this point). It was reported that the fecundity of *A. pisum* reared on artificial diets decreased sharply till sterility, which might be resulted from the decrease in the number of their symbiotic bacteria^53^. Our finding that the effects of the artificial diet on the development and fecundity were distinctly different in different aphids suggested that the nutritional requirement was quite different for different aphids. On the other hand, our results indicated that 90% of cotton plant-reared aphids emitted cornicle droplets when disturbed, while only approximately 60% of diet-reared and antibiotic-treated aphids emitted droplets. As the antibiotic-treated aphids were also reared on diet, the emission proportion of this treatment was not significantly different from that of normal diet-reared aphids. Thus, it is most likely that the lower proportion of droplet emission in diet-reared aphids was an effect of the diet, which might lack some important ingredients involved in the biosynthesis of EβF. It was previously found that the total amount of EβF in diet-reared aphids could be increased by adding acetate in the diet^43^. Here, our data showed that the weight of antibiotic-treated aphids reduced by approximately 50% compared with that of the untreated aphids, and the concentration of EβF (ng per aphid) in the antibiotic-treated aphids was also less than that in both plant- and diet-reared aphids. One possible explanation for this may be that removal of the obligate endosymbionts could negatively affect the development of the aphids, and the *ispA* gene of *Buchnera* might also play a positive role in the process of EβF biosynthesis by providing the precursor FPP for the host aphid.

EβF as the major component of the alarm pheromone of many aphid species plays important roles in mediating multitrophic interactions among aphids, host plants, and aphid natural enemies^11^, which has been used as a repellent against aphids or an attractant for natural enemies of aphids. Although plant-originated EβF synthase genes have been engineered into crop plants which emitted EβF and repelled aphids^32^, identification of the genes responsible for the biosynthetic pathway leading to production of EβF in aphids is certainly significant as these genes are at the core of the molecular mechanism underlying EβF biosynthesis. With these genes, we can not only synthesize the “real” aphid alarm pheromones *in vitro*, but also control aphid behaviors by genetic modification. Now that we excluded the possibility of host plants and obligate endosymbionts as the sources for biosynthesis of aphid alarm pheromone, what can we do next to identify the genes responsible for the synthesis of alarm pheromone in aphids? FPPS is a key enzyme in the isoprenoid pathway, supplying C_15_ precursors for several classes of essential metabolites in many organisms, including dolichol, ubiquinone, sesquiterpene and sterol^12^. As shown in plants, FPP is the precursor for EβF synthesis^11^. The challenge now is to identify the enzyme(s) responsible for converting FPP to EβF in aphids. The plasticity of isoprenoid synthases including FPPS has been demonstrated in different organisms. It was repeatedly reported that recombinant FPPSs from aphid species also function as GPPS (with dual FPP/GPP synthase activity)^19–23^. Similarly, GPPS from *Ips* beetles could not only synthesize GPP from DMAPP and IPP, but also convert GPP to the monoterpene myrcene^41^. Now that the whole genome sequence of aphid is available and homology cloning of EβF synthase gene from aphids is unsuccessful, the emphasis for further work will be on the multifunctionality of isoprenoid synthases. The RNA interference technique and transcriptome sequencing would be useful in this line of work.

## Methods

### Aphids and artificial diet

*A. gossypii* and *M. persicae* have been maintained on potted cotton and sweet pepper plants (20 ± 0.5 °C, L16:D8) in China Agricultural University, Beijing since 2008. The artificial diets for rearing aphids were prepared according to the published recipe^44^, but cholesterol was omitted from the diet since we found it was not ideal for aphid development and a previous study showed that aphids could grow from larval stage to adults on a synthetic diet without sterol^54^. The basal composition for diet preparation was given in Supplementary Table S1. Various amino acid stock solutions were separately prepared and then used to mix with trace-metal miscible liquids, ascorbic acid, biotin, folic acid and vitamin B group (pH7.0, adjusted with 30% KOH). The final mixture was sterilized by passing through a filter (Acrodisc^®^ Syringe Filters with Supor^®^ Membrane, 0.2 μm, Life Sciences), then distributed into 1.5 mL sterile centrifugal tubes and stored at −20 °C for further use.

### Artificial rearing of aphids

A two-way glass tube (2.5 cm × 3.0 cm) with the diet (150–200 µL) sealed between two layers of taut parafilm at one end was used as the feeding device. The parafilm was sterilized with ultraviolet light. Forty adult aphids were introduced into each tube, and then the bottom of the tube was covered with a filter paper and a rubber band to prevent escape of aphids. The tube was placed in a petri dish (Φ9.0 cm) with a layer of wet filter paper for keeping humidity. The adults were removed the next day but the newly-born nymphs were maintained in the tube. The diet was replaced every 2 days until the aphids reached maturity.

### Effects of the artificial diet on the growth and development of aphids

One newly-born aphid was introduced into the feeding tube containing the artificial diet. The exuvial case of aphid was checked every 5 h during the nymphal stage, and the duration of each stage and the number of offspring per aphid on artificial diet was recorded. As a control, the developmental duration and fecundity of aphids on plants were also observed by using round-leaf method: 1% agar gel was poured into a petri dish (Φ3 cm), and when the gel became solidified, a round pepper or cotton leaf was laid on the gel for rearing the green peach aphid and the cotton aphid, respectively. One newly-born aphid was then introduced into each dish, with 20 replicates.

### Elimination of *Buchnera* by antibiotic treatment

Fifty newly-born aphids were moved into the feeding tube with the artificial diet containing 100 μg/mL antibiotics (tetracycline and rifampicin, respectively). After fed for 2 d, 6 d, 10 d, 12 d or 14 d, the aphids were transferred to diet without antibiotics, and samples were taken separately for molecular detection of *Buchnera* after 5 or 10 additional days, with three replicates. The control group was consistently fed with artificial diet without antibiotics. The developmental durations and fecundity of aphids with and without antibiotic treatment were measured during different stages by using the feeding tube method as described above, with 20 replicates.

### Molecular detection of *Buchnera*

Five aphids from each treatment were subjected to DNA extraction for molecular detection. DNA extraction was performed by using the potassium acetate method as described^55^. *Buchnera* was detected in aphids according to the previously reported method^56^. The primers used were listed in Supplementary Table S2. The primers targeting the *ispA* gene of *Buchnera* were designed to specifically ensure that the *ispA* gene that is suspected to be involved in the biosynthesis of EβF is absent after antibiotic treatment. As the *ispA* sequence was unavailable for *A. gossypii*, the genome sequences of *Buchnera aphidicola* from other aphids (*e.g*., *Acythosiphon pisum*, *M. persicae*, *Schizaphis graminum* and *Aphis glycines*) were retrieved from NCBI and degenerate primers (Buchisp-F/R) were designed for PCR amplification of *A. gossypii ispA*. The PCR product was then cloned and sequenced according to conventional protocol. Based on the obtained *ispA* sequence, the primers (C-*ispA*-F/R) were thus designed to detect *Buchnera* in *A. gossypii*.

### Analysis of volatile releases from aphids by SPME and GC-MS

Solid-phase micro-extraction (SPME) combined with gas chromatography-mass spectrometer (GC-MS) was performed for analysis of the volatile releases from plant-reared, diet-reared and antibiotic-treated diet-reared aphids. A synthetic farnesene solution (provided by Group of Chemical Ecology of Forest Insects, Institute of Zoology, Chinese Academy of Sciences) was diluted 20 times with acetone and used as the standard, and 10 μL standard solution was transferred to a glass tube adapted to the SPME with pipette for GC-MS analysis. The standard solution was first maintained for 20 min at 30 ± 0.2 °C, and then the volatile ingredients were sampled for 30 min with 100-µm polydimethyl-siloxane SPME fibers (Supelco^®^, Sigma, USA) and immediately analyzed by GC-MS on Perkin Elmer Clarus600. One or ten aphids were crushed with an insect needle prepared from a glass tube and the volatile releases were collected with SPME and then analyzed by GC-MS under the following conditions: The temperature of injection port was from 60 °C to 250 °C at the speed of 200 °C/min. Samples were purged with helium at 4 ml/min for 10 min to make the absorbed substances totally volatilized. The initial temperature of pillar was 100 °C, kept for 1 min, then changed to 180 °C at 6 °C/min and to 280 °C at 15 °C/min, kept for 10 min. Mass spectra were obtained in the EI mode at 80 *eV* (scanned mass range from 45 to 300 *amu*) and analyzed with TurboMass Ver5.4.2. Each treatment was repeated for 3 times.

### Quantitative analysis of cornicle secretions

The propensity of aphids to emit cornicle droplets was examined for the fourth instars reared on cotton plants (*N* = 235, 239, 215, 279), artificial diet (*N* = 407, 369, 271, 355) and antibiotic-treated aphids (*N* = 393, 318, 360, 275). The methods of Mondor *et al*.^57^ and Fischer and Lognay^58^ were combined for the quantitative analysis of cornicle secretions. The aphid was stimulated to produce droplet from the cornicle by pressing the dorsum gently with an insect pin under a dissecting microscope. When the cornicle fluid was emitted, the aphid with secretion was put in a 2-ml centrifuge tube (on the ice) that contained 200 μl of distilled hexane and 0.2 μl decane (purity >99.9%) as an internal standard for quantification. A stir bar was placed inside the centrifuge tube and placed onto a working magnetic stir plate for 10 min to completely crush the aphid and extract EβF. Then, 180 μl of clean supernatant was isolated from the homogenate by using a centrifuge and transferred to a chromatography vial. Samples were maintained at −20 °C until analysis. One microliter of sample was injected in Perkin Elmer Clarus 600 for GC-MS analysis by using the methods described^57, 58^. If an aphid did not produce any cornicle fluid, it was placed in another 1.5-ml centrifuge tube for weighting. At least 100 aphids were collected for each tube, with 4 replicates. The numbers of aphids emitting or not emitting cornicle droplets were recorded.

### Statistical analyses

Developmental duration and fecundity data of plant-reared, diet-reared, and antibiotic-treated diet-reared aphids were analyzed by using least significant difference (LSD) multiple comparison method at 5% and 1% levels on SPSS 20.0 software. The EβF amounts were calculated by the peak areas of EβF and decane on TurboMass Ver.5.4.2 software. A covariance analysis of the EβF amounts per mg of aphid and per aphid reared under different conditions was also conducted.

## Electronic supplementary material

Supplementary info
