# Supplementary material for: Host plants and obligate endosymbionts are not the sources for biosynthesis of the aphid alarm pheromone
Source: Sci Rep. 2017 Jul 20;7:6041. doi: 10.1038/s41598-017-06465-9 (PMC5519760; doi:10.1038/s41598-017-06465-9)
Supplement: Supplementary file 1 — Supplementary info [file 41598_2017_6465_MOESM1_ESM.pdf]

Host plants and obligate endosymbionts are not the sources for biosynthesis of the aphid alarm pheromone

Zhi-Juan Sun, Zheng-Xi Li[†]

Department of Entomology, China Agricultural University, 2 Yuanmingyuan West Road, Beijing 100193, China

[*]Author for correspondence: Dr. Zheng-Xi Li, Department of Entomology, ChinaAgriculturalUniversity, 2 Yuanmingyuan West Road, Beijing 100193, China. Tel/Fax: +86 10 62733608; E-mail: zxli@cau.edu.cn.

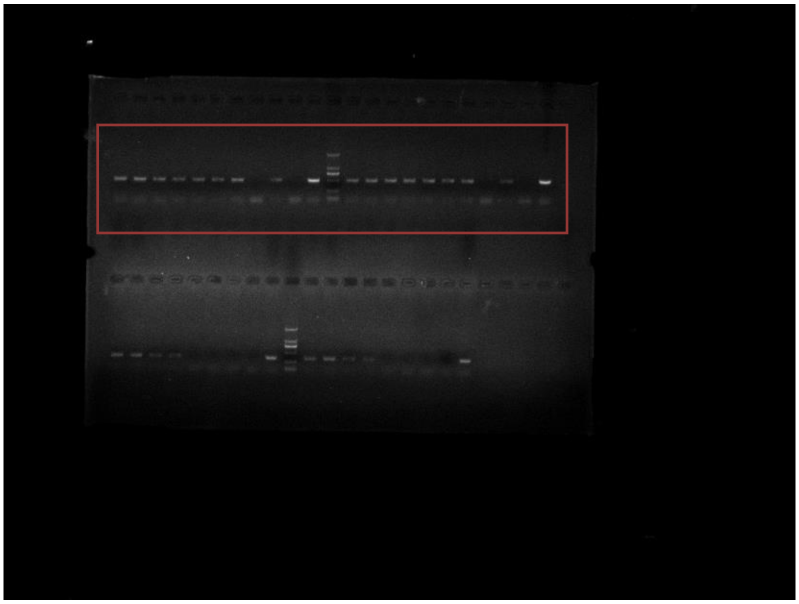

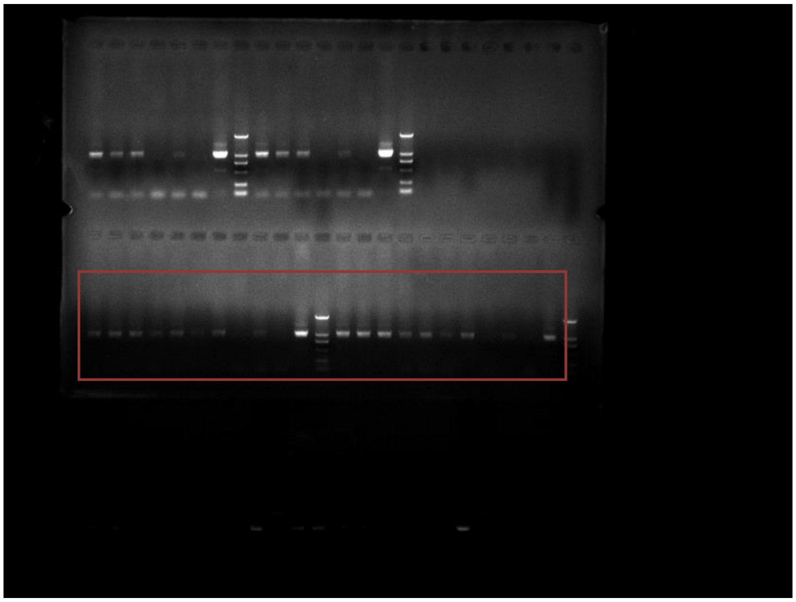

Supplementary Figure S1. Full-length gels corresponding to **Figure 1** (cropped): argS-rrs-F/R (upper) and M-ispA-F/R (down).

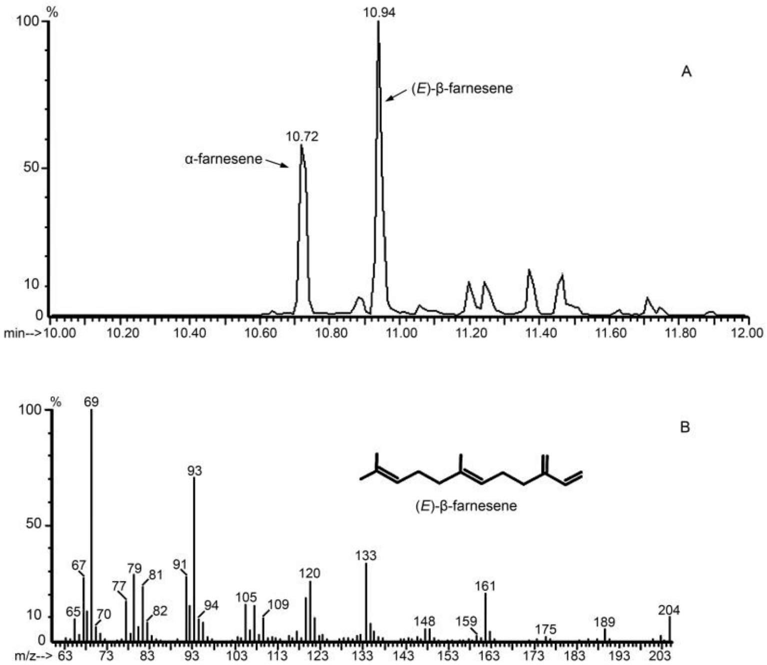

Supplementary Figure S2. Analysis of standard farnesene solution by GC-MS. A: The retention times for (*E*)-β-farnesene and α-farnesene are 10.94 and 10.72, respectively. B: Mass spectrum for EβF (with a molecular weight of 204).

Supplementary Table S1. Composition of the artificial diet for rearing aphids (50 mL)

Ingredient	Dosage/mg	Ingredient	Dosage/mg
L-Alanine	100.0	L-Tyrosine	20.0
L-Arginine	135.0	L-Valine	40.0
L-Asparagine	275.0	Ascorbic acid	50.0
L-Aspartic acid	70.0	KH_2PO_4	250.0
L-Cysteine	20.0	$MgCl_2.6H_2O$	100.0
L-Glutamic acid	70.0	Sucrose	7500.0
L-Glutamin	75.0	$CuCl_2 \cdot 2H_2O$	0.069
Glycine	40.0	$MnCl_2 \cdot 4H_2O$	0.130
L-Histidine	40.0	$ZnSO_4 \cdot 7H_2O$	0.139
L-Isoleucine	40.0	$FeCI_3 \cdot 3H_2O$	0.334
L-Leucine	40.0	Thiamin (VB_1)	1.25
L-Lysine	60.0	Nicotinic acid (VB_3)	5.0
L-Methionine	40.0	Pyridoxol (VB_6)	1.25
L-Phenylalanine	20.0	Calcium pantothenate (VB_5)	2.5
L-Proline	40.0	Biotin (VB_7)	0.05
L-Serine	40.0	Inose	25.0
L-Threonine	70.0	Choline chloride	25.0
L-Tryptophane	40.0	Folic acid	0.25

Supplementary Table S2. Primers used in this study

**Primer name**	**Sequence (5'→3')**	T_a /°C	**Expected size (bp)**
Buchisp-F	ATGAGYATKTAYTCTACTAGTCART	45	1347
Buchisp-R	CAYARTAAATTRTCAAAHGGDGT		
argS-rrs-F	TATGATTTGCTCTAATTACATTATG	53	1033
argS-rrs-R	CTAATCTCGTCTGGGTTCATC		
M-*ispA*-F	AAAGCTAATCCAATAGAACTAGAA	53	511
M-*ispA*-R	CTTGTTTAGTGTATACTACTGGAAA		
C-*ispA*-F	TGATGATTTACCGTCCATTGATA	55	344
C-*ispA*-R	TTTGAAGATAAATATGGCAATCG		
